# Impact of the β-Lactam Resistance Modifier (−)-Epicatechin Gallate on the Non-Random Distribution of Phospholipids across the Cytoplasmic Membrane of *Staphylococcus aureus*

**DOI:** 10.3390/ijms160816710

**Published:** 2015-07-23

**Authors:** Helena Rosado, Robert D. Turner, Simon J. Foster, Peter W. Taylor

**Affiliations:** 1 School of Pharmacy, University College London, London WC1N 1AX, UK; E-Mail: h.rosado@ucl.ac.uk; 2 Krebs Institute, University of Sheffield, Western Bank, Sheffield S10 2TN, UK; E-Mails: r.d.turner@sheffield.ac.uk (R.D.T.); s.foster@sheffield.ac.uk (S.J.F.)

**Keywords:** epicatechin gallate, *Staphylococcus aureus*, membrane phospholipids, membrane asymmetry, beta-lactam susceptibility, bacterial cell division

## Abstract

The polyphenol (−)-epicatechin gallate (ECg) inserts into the cytoplasmic membrane (CM) of methicillin-resistant *Staphylococcus aureus* (MRSA) and reversibly abrogates resistance to β-lactam antibiotics. ECg elicits an increase in MRSA cell size and induces thickened cell walls. As ECg partially delocalizes penicillin-binding protein PBP2 from the septal division site, reduces PBP2 and PBP2a complexation and induces CM remodelling, we examined the impact of ECg membrane intercalation on phospholipid distribution across the CM and determined if ECg affects the equatorial, orthogonal mode of division. The major phospholipids of the staphylococcal CM, lysylphosphatidylglycerol (LPG), phosphatidylglycerol (PG), and cardiolipin (CL), were distributed in highly asymmetric fashion; 95%–97% of LPG was associated with the inner leaflet whereas PG (~90%) and CL (~80%) were found predominantly in the outer leaflet. ECg elicited small, significant changes in LPG distribution. Atomic force microscopy established that ECg-exposed cells divided in similar fashion to control bacteria, with a thickened band of encircling peptidoglycan representing the most recent plane of cell division, less distinct ribs indicative of previous sites of orthogonal division and concentric rings and “knobbles” representing stages of peptidoglycan remodelling during the cell cycle. Preservation of staphylococcal membrane lipid asymmetry and mode of division in sequential orthogonal planes appear key features of ECg-induced stress.

## 1. Introduction

The heterogeneous nature of eukaryotic membranes is well established. Membrane proteins that mediate essential functions such as signal transduction and protein secretion are laterally segregated into microdomains rich in lipid bilayer constituents, such as sphingolipids and sterols [1,2]. The localization of protein complexes in these restricted membrane lipid rafts has profound importance for cell function [3] and cellular functions are also strongly influenced by the different biophysical properties of the outer and inner surfaces of the membrane bilayer as a consequence of the non-random distribution of amphipathic components across the lipid palisade. Maintenance of the asymmetric distribution of phospholipids between the inner and outer leaflets of the plasma membrane is an energy-dependent process imposing a high metabolic burden on the cell [4,5]. Transport of phosphatidylserine from the inner leaflet to the cell surface provides a signal for removal by phagocytosis of apoptotic cells [6] and promotes many other cellular functions [6,7,8,9].

Phospholipid compartmentalization has been less extensively investigated in prokaryotic membranes. Studies with lipid-binding fluorescent dyes have revealed that phospholipids may form microdomains within the cytoplasmic membrane (CM); for example, the poles and septa of Gram-negative [10] and Gram-positive [11] bacteria are rich in cardiolipin (CL), whereas phosphatidylglycerol (PG) displays a helical distribution within the bilayer of *Bacillus subtilis* [12]. Phospholipid microdomains play a crucial role in the localization and function of bacterial membrane proteins and protein complexes, and their disruption impairs key cell functions such as biofilm formation and protein secretion [13]. In addition, dynamic structural and functional heterogeneity of membrane-embedded bacterial proteins is partially controlled by changes in the composition of the local phospholipid environment [14]. Although it is well established that the outer membrane of Gram-negative bacteria is highly asymmetric with respect to lipid distribution, with lipopolysaccharide located exclusively in the outer and phospholipid in the inner leaflet [15], few attempts have been made to determine phospholipid distribution across the prokaryotic CM. The realization that changes to the radius of membrane curvature, facilitation of septum formation during division of rod-shaped bacteria and migration of phospholipids across the bilayer would necessitate phospholipid asymmetry within the CM prompted a search for translocators with the capacity to transfer phospholipids across the bilayer [16]: a number of translocases of the ATP binding cassette (ABC) transporter superfamily have been implicated in the outward movement of bacterial phospholipids [17] but the nature and degree of asymmetry was not investigated in these studies.

*Staphylococcus aureus* is a common component of the mammalian microbiota, but it also causes severe nosocomial and community acquired infections [18,19]. The potential of these opportunistic pathogens to cause life-threatening infections is exacerbated by their capacity to accumulate antibiotic resistance genes and a large proportion of clinical isolates are now resistant to the entire arsenal of β-lactam antibiotics [20], agents that historically have been the primary defence against staphylococcal infections. The staphylococcal CM is, unusually, composed predominantly of PG, LPG (PG modified by enzymatic transfer of a lysine residue to the polar head group) and CL [21,22,23]. Recent studies have demonstrated a high degree of asymmetry between inner and outer leaflets of the bilayer [22,24]. Further, both *S. aureus* mutants resistant to the platelet cationic peptide tPMP-1 [22] and sequential *S. aureus* clinical isolates with decreased levels of susceptibility to the membrane-active antibiotic daptomycin [24] displayed increased rates of translocation of positively charged LPG from the inner to the outer leaflet of the membrane. The resulting increased net positive charge at the staphylococcal surface due to changes in the degree of asymmetry appear to be directly responsible for reduced susceptibility to these bactericidal agents.

β-lactam resistant isolates of *S. aureus* (MRSA) avoid killing by penicillins and cephalosporins due to acquisition of *mecA* or its homologue *mecC*, genes encoding penicillin binding protein (PBP) 2a, a transpeptidase insensitive to β-lactam agents that facilitates continued peptidoglycan synthesis in the presence of otherwise inhibitory β-lactam drug concentrations [25,26]. PBP2a and its partner protein, PBP2, a multifunctional enzyme whose transpeptidase domain is inactivated by active-site β-lactam acylation, form part of the staphylococcal cell division machinery located mid-cell within a divisome of over twenty proteins [27]. We have established that catechin gallates, naturally occurring polyphenolic membrane intercalating agents [28], have the capacity to reversibly abrogate β-lactam resistance in MRSA by dissipation of key divisome components from the septal site of cell division [23,29]. The most potent of these, (−)-epicatechin gallate (ECg), penetrates deep into the hydrophobic core of the CM (30), eliciting major changes in the thermotropic behaviour of the bilayer and inducing a comprehensive reconfiguration of membrane architecture as the bacteria respond to ECg membrane intercalation [23,30]. Although ECg-modified cells remain viable, alterations to the lipid palisade provide a suboptimal environment in which the cell division machinery is forced to operate, leading to disruption of the close physical and functional relationship between PBP2 and PBP2a that is essential for β-lactam resistance [29,30]. To determine the impact of ECg on the molecular configuration of the CM, we examined the distribution of LPG, PG and CL following intercalation of the polyphenol into the bilayer; we also used atomic force microscopy to assess the effect of CM reconfiguration on the orthogonal mode of cell division [31]. We conclude that the EMRSA-16 CM is highly asymmetric; intercalation of ECg has a small but significant impact on the distribution of lipids across the bilayer and division of ECg-exposed EMRSA-16 continues to occur in orthogonal planes. 

## 2. Results

### 2.1. Intercalation of ECg into the EMRSA-16 CM Induces Major Changes to the Bacterial Phenotype

**Figure 1:**
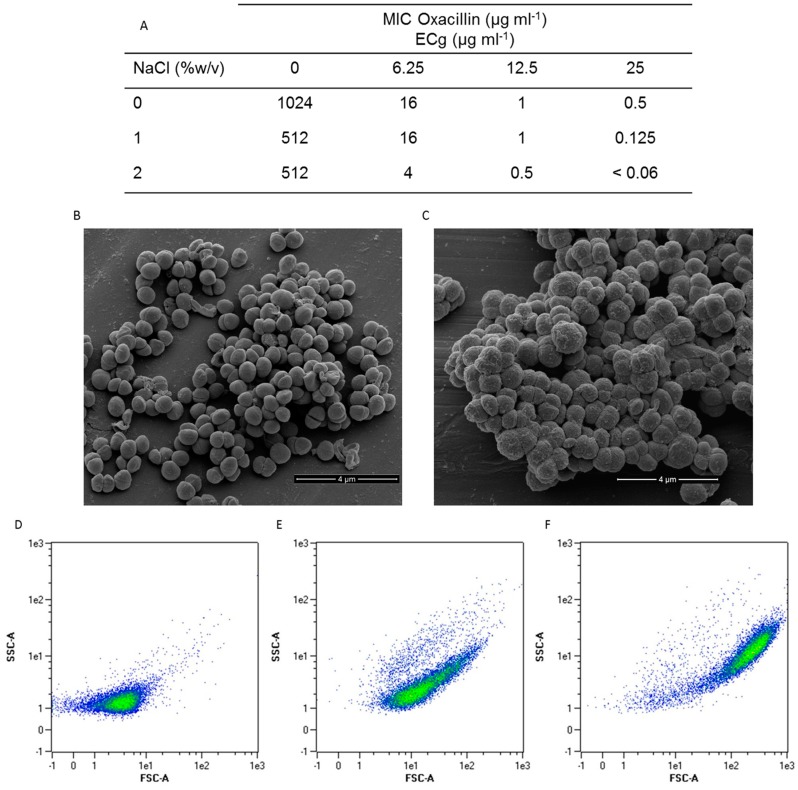
ECg induces reversible changes to the EMRSA-16 phenotype. ECg abrogates β-lactam (oxacillin) resistance in Mueller-Hinton (MH) broth and NaCl-supplemented MH broth (**A**) Scanning electron microscopy reveals that incorporation of 12.5 µg/mL ECg into growth medium alters the appearance of the staphylococcal cells (**C**) in comparison to control cells grown in the absence of the compound (**B**). Flow cytometric analysis (10,000 events) of EMRSA-16 grown in the absence (**D**) or presence of 12.5 µg/mL ECg for 1 h (**E**) or 4 h (**F**) shows increases in size (FSC; forward scatter) and changes to cell granularity (SSC; side scatter) as a consequence of exposure to the compound.

Exposure of EMRSA-16 to ECg has a profound impact on susceptibility to oxacillin (Figure 1A) and other β-lactam agents [32], in both the presence and absence of NaCl; the contingent level of β-lactam resistance is considered to be increased by addition of salt to the MIC test medium [33]. As previously noted [34,35] ECg induces multicellular aggregates, indicating poor separation of daughter cells following division (Figure 1B,C). The clumps of partially divided cocci appear larger and display a rougher surface than control cells. Progressive increase in size over a 4 h incubation period was confirmed by flow cytometry and increases in cell wall thickness [34] and roughness of the cell surface were reflected in temporal increases in side scatter fluorescent signals, illustrative of changes in geometry and internal structure (Figure 1D–F). These changes were not accompanied by significant alteration of the growth of EMRSA-16 as determined by optical density measurements.

### 2.2. Intercalation of ECg into the EMRSA-16 CM Induces Small but Statistically Significant Changes in Membrane Lipid Symmetry

**Figure 2:**
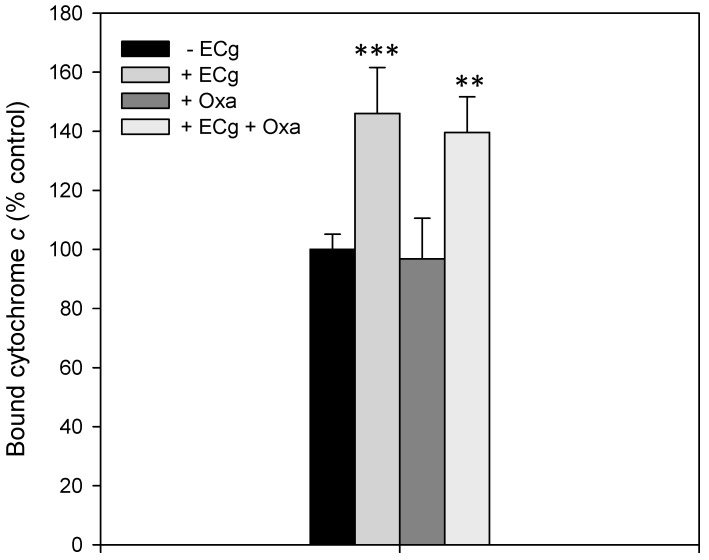
Growth of EMRSA-16 in MH broth containing 12.5 µg/mL ECg increases surface binding of the cationic protein cytochrome c. ECg-exposed and control cells were suspended (OD_600_ 7.0) in 3-(*N*-morpholino)propanesulfonic acid (MOPS) buffer (20 mM, pH 7.0) at 37 °C for 10 min together with 0.5 mg/mL cytochrome c. The remaining (unbound) cytochrome c in the supernatant was quantified spectrophotometrically at 530 nm and compared to a MOPS buffer control (equivalent to 100% unbound). In some experiments 1 µg/mL oxacillin was added to the growth medium. Each experiment was repeated a minimum of five times, each on different days. Student’s one-tailed *t*-test: ** *p* < 0.01; *** *p* < 0.001; compared with control (−ECg).

Growth for 4 h of EMRSA-16 in MH broth containing ECg increased the overall surface charge by approximately 1.5-fold, as determined by the amount of cytochrome c bound to the staphylococcal cells. The presence of 1 µg/mL oxacillin had no impact on cytochrome c binding to the staphylococcal cells (Figure 2). As the orientation of negatively charged PG and CL and positively charged LPG play a key role in the determination of the overall charge profile of the staphylococcal surface [22], we determined the distribution of the three major staphylococcal phospholipids across the EMRSA-16 CM bilayer and investigated the impact of growth in the presence of ECg on lipid orientation.

There have been few attempts to determine membrane lipid asymmetry in prokaryotes. Mukhopadhyay *et al.* [22] adapted methods from studies of eukaryotes to determine lipid distribution between the inner and outer leaflets of the *S. aureus* CM using fluorescently labeled lipid probes in combination with flow cytometric analysis of probe orientation. As a range of physical and biological parameters are known to influence the behavior of lipid probes within membranes [36,37], we optimized fluorescent lipid distribution procedures by evaluating key parameters of probe incorporation into membranes.

Various concentrations of the PG analogue, 18:1–06:0 NBD-PG, were incubated with either 10^6^ or 10^7^ CFU/mL EMRSA-16 for up to 40 min (Figure S1). In both cases the fluorescence intensity increased over the period of incubation using probe concentrations ranging from 0.1 to 10.0 µM. Fluorescence intensity also continuously increased when concentrations as high as 50 µM were used. High concentrations of NBD-lipids impacted on cell viability and the degree of incorporation of probe into the membrane was markedly dependent on the probe concentration; we therefore conducted all further experiments using 10 µM probe and 10^6^ CFU/mL. The nature of the fatty acid chains in the PG analogues influenced the rate and degree of incorporation into the CM (Figure S2). Short chain 06:0–06:0 NBD-PG readily intercalated into the CM reaching maximum fluorescence intensity almost immediately (Figure S3) but the overall degree of incorporation was low (Figure S4), likely to be a reflection of its low hydrophobicity compared to natural membrane phospholipids. Long chain 18:1–06:0 NBD-PG intercalated more slowly but more extensively into the membrane. Its higher degree of hydrophobicity compared to 06:0–06:0 NBD-PG appears to permit prolonged CM intercalation at a relatively slow rate. In contrast to 18:1–06:0 NBD-PG, bilayer intercalation of 06:0–06:0 NBD-PG did not permit visualization of the probe by fluorescence imaging (Figure S2). The CL analogue readily intercalated the membrane (Figure S3), but the overall incorporation was low and comparable to 06:0–06:0 NBD-PG (Figure S4). The two long fatty acid chains confer a higher level of hydrophobicity to the lipid, but the NBD fluorophore is attached to the glycerol moiety and this may interfere with CM intercalation. Growth of the bacteria in the presence of 12.5 µg/mL ECg for 4 h had little impact on CM incorporation of the probes (Figures S3 and S4). The rate (Figure S5) and the extent (Figure S6) of NBD-lipid incorporation into the CM were greater at RT compared to 4 °C, a reflection of the fluid state of the CM under these different conditions. Intercalation of 06:0–06:0 NBD-PG was less temperature dependent than that of 18:1–06:0 NBD-PG. Prior incorporation of ECg into the bilayer had no significant impact on probe incorporation (Figures S5 and S6). These data indicate that membrane lipid asymmetry may be efficiently determined using a cell density of 10^6^ and a fluorescent probe concentration of 10 µM at RT for 30 min.

**Figure 3:**
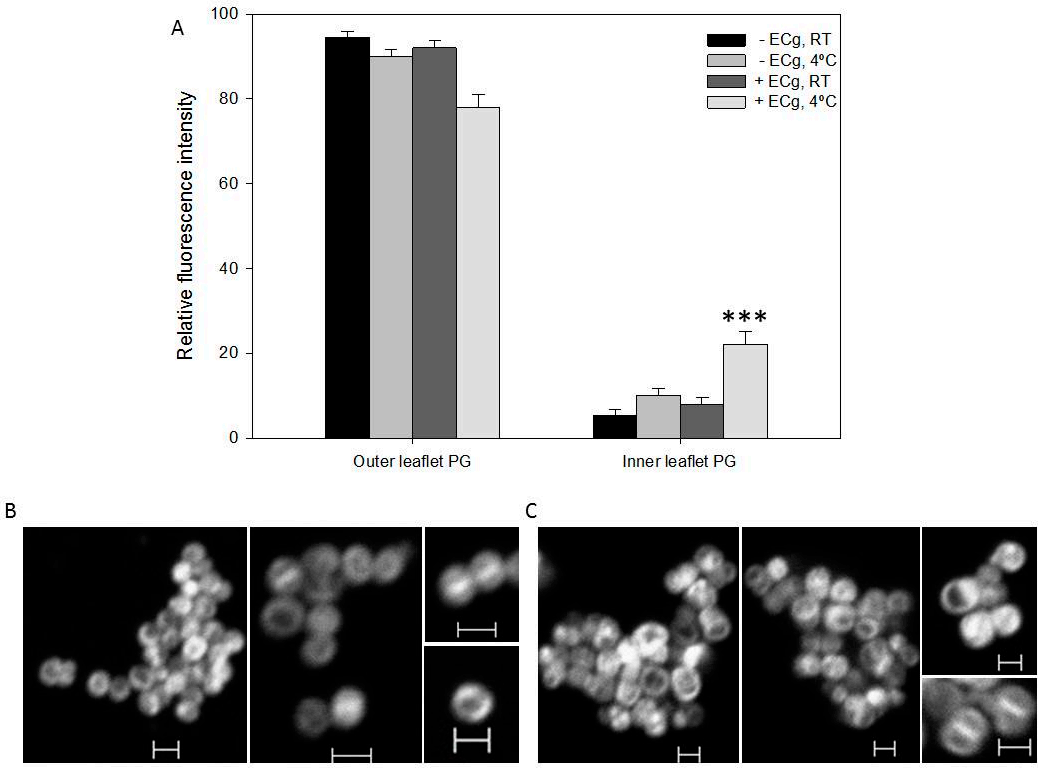
ECg elicits small but significant changes to the distribution of PG across the EMRSA-16 CM. (**A**) relative fluorescence intensity (±1 SD) of 18:1–06:0 NBD-PG labelled cells grown in the absence or presence of 12.5 µg/mL ECg. Fluorescence emission was measured after 30 min exposure to the lipid analogue and following quenching with 25 mM sodium dithionate at room RT and 4 °C (*n* = 5). *** *p* < 0.001; two-tailed Student’s *t*. Fluorescence determined by flow cytometry (10,000 events collected). CLSM of EMRSA-16 grown in the absence (**B**) and presence (**C**) of 12.5 µg/mL ECg; cells were labeled with 10 µM 18:1–06:0 NBD-PG at RT. Scale bars 1 µm.

The use of fluorescent derivatives of membrane phospholipids is based on strong evidence that, at equilibrium, the analogues are distributed across the bilayer in similar fashion to the native lipid species due to enzyme-catalysed transbilayer lipid movement [17,36,37,38,39,40]. The procedure for quantification of PG in the inner and outer leaflets of the CM relies on the capacity of sodium dithionite to quench outer, but not inner, leaflet fluorescence of incorporated probe. We determined that fluorescence quenching of NBD-lipids occurred immediately after addition of sodium dithionite and remained constant for at least 7 min; dithionite quenching was therefore sustained for 5 min followed by immediate determination of residual inner leaflet fluorescence intensity. The distribution of 18:1–06:0 PG was highly asymmetrical, with over 90% in the outer leaflet (Figure 3A) when determined at RT. We also determined the distribution at 4 °C to allow a more direct comparison with previously published data [22]; the fluorescence emission was comparable at these two temperatures. Cultivation of EMRSA-16 in the presence of ECg had a small but significant impact of the degree of PG asymmetry (Outer leaflet: *p* = 0.00032 at RT; *p* = 0.00068 at 4 °C. Inner leaflet: *p* = 0.00032 at RT; *p* = 0.00068 at 4 °C). The degree of asymmetry of control cells at 4 °C was essentially identical to that reported by Mukhopadhyay *et al.* [22] using strains derived from *S. aureus* ISP479 [41]. Confocal laser scanning microscopy (CLSM) revealed no significant ECg-induced differences in the CM distribution of 18:1–06:0 PG (Figure 3B,C).

**Figure 4:**
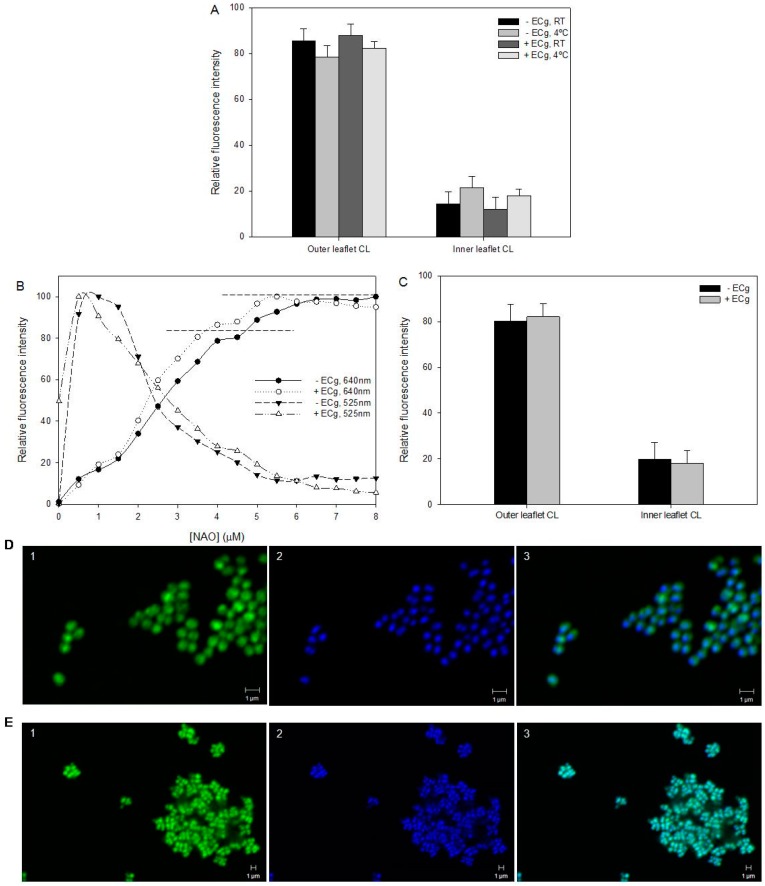
CL is asymmetrically distributed between the outer and inner leaflets of the EMRSA-16 CM and is unaffected by intercalation of ECg into the bilayer. (**A**) Relative fluorescence intensity of 18:1–18:1 NBD-CL labelled EMRSA-16 in the absence or presence of 12.5 µg/mL ECg. Fluorescence emission was determined after 30 min exposure at RT and 4 °C to the lipid analogue followed by dithionate quenching (*n* = 5). RT, room temperature; (**B**) Relative fluorescence intensity of CL in the EMRSA-16 CM in the absence (closed symbols) or presence (open symbols) of 12.5 µg/mL ECg, as determined by NAO titration. Fluorescence emission was read at 525 nm (decreasing fluorescence) and at 624 nm (increasing fluorescence) after 20 min cell exposure to the dye at 4 °C. NAO binds initially to outer leaflet CL until saturation at approximately 4–4.5 µM NAO, and then to the inner leaflet CL until complete saturation at approximately 5.5 µM NAO. The transition is marked by horizontal lines; (**C**) Quantification of CL present in the outer and inner leaflets of the EMRSA-16 CM (*n* = 5). No significant differences between control and ECg-grown bacteria with either method: *p* > 0.1; two-tailed Student’s *t*; CLSM of EMRSA-16 in the (**D**) absence and (**E**) presence of 12.5 µg/mL ECg; cells labelled with (1) 5 µM NAO, (2) 5 µM Hoescht 33342 and images merged (3). Scale bars 1 µm.

**Figure 5:**
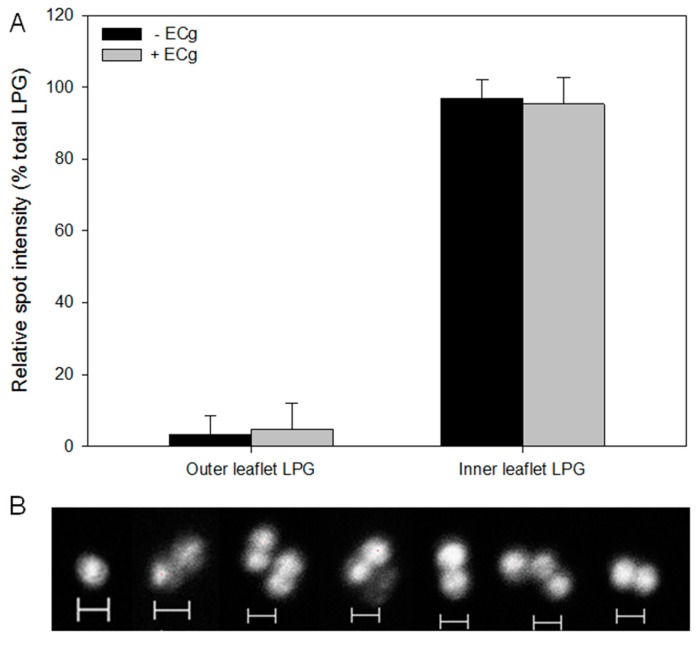
The degree of LPG asymmetry of the EMRSA-16 CM is unaffected by intercalation of ECg into the bilayer. (**A**) Relative LPG content of the outer and inner leaflets of the CM in the presence or absence of 12.5 µg/mL ECg (*n* = 5). No significant difference between control and ECg-grown bacteria was found: *p* > 0.1; two-tailed Student’s *t*. Outer leaflet LPG was quantified by measurement of membrane-bound fluorescamine, which does not cross the bilayer; inner leaflet LPG was quantified with ninhydrin after lipid separation on 2D-HPTLC plates; (**B**) CLSM of EMRSA-16 labelled with fluorescamine. Scale bars 1 µm.

The two methods used to determine bilayer distribution of CL yielded comparable data. Using 18:1–18:1 NBD-CL (Figure S7 and Figure 4A) and the fluorescent dye 10-*N* nonyl acridine orange (NAO) in tandem with flow cytometry (Figure 4B), we determined that ~80% of the CL analogue resided in the outer leaflet of the EMRSA-16 CM (Figure 4C). NAO binds initially to phosphate groups in the outer leaflet of the bilayer and binds only to the inner leaflet after saturation of the outer surface. Growth in ECg-containing medium had no significant effect on the degree of asymmetry. CLSM of ECg-grown and control EMRSA-16 exposed to NAO revealed increased fluorescence at the division septum, suggestive of accumulation of anionic lipids, particularly CL, at this location (Figure 4D,E). LPG is largely confined to the inner leaflet of the CM, with only 3%–5% in the outer leaflet and no significant difference was found in LPG across the lipid palisade between control and ECg-grown EMRSA-16 (Figure 5A). Both CL and LPG values are similar to those obtained by Mukhopadhyay *et al.* [22]. Due to the predominantly inner leaflet location of LPG, fluorescamine bound to the cell surface to a limited extent. Nevertheless, we detected accumulation of LPG at the division septum using CLSM, in similar fashion to the distribution of the anionic phospholipids PG and CL (Figure 5B).

### 2.3. ECg Does Not Alter the Mode of Peptidoglycan Biosynthesis or Septum Formation in EMRSA-16

**Figure 6:**
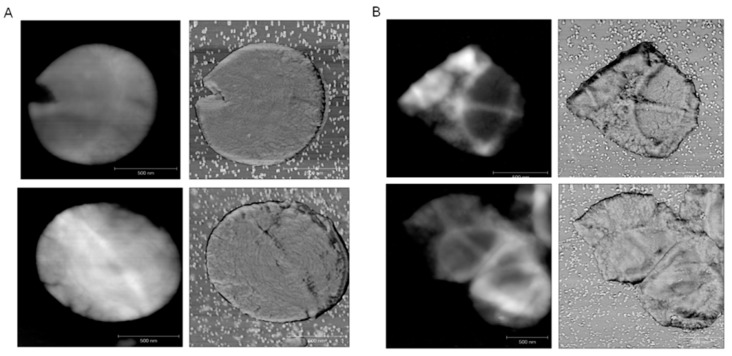
AFM images of sacculi prepared from EMRSA-16 grown in the absence (**A**) and presence (**B**) of 12.5 µg/mL ECg. AFM height (**left** panel) and phase (**right** panel) images are shown. Scale bars 500 nm. All images show “piecrusts”, thickened bands of peptidoglycan encircling intact cells that represent the most recent plane of division, and less distinct “ribs” indicative of previous sites of orthogonal cell division. Concentric rings associated with nascent peptidoglycan are illustrated in one control phase image; these structures may be remodelled by autolysins to form “knobbles”, seen in the upper phase image of peptidoglycan from ECg-exposed cells. A detailed description of peptidoglycan architecture during the division cycle can be found in Reference [31].

Intercalation of ECg into the CM partially delocalizes PBP2 from the septal site of cell division [23] and disrupts functional and physical associations between PBP2 and PBP2a within the divisome [29], suggesting that ECg may engender sites, remote from the septum, where continued peptidoglycan synthesis is taking place. This should manifest as changes to the equatorial, sequential orthogonal mode of cell division typical of *S. aureus* [31]. We therefore examined the planes of cell division in EMRSA-16 using AFM, comparing cells grown for 4 h in 12.5 µg/mL ECg with control cells (Figure 6). The topography of the sample is revealed by the height signal and the phase signal provides insights into adhesive and mechanical aspects in addition to topographic features. In controls, the cell wall displays a characteristic architecture: as the peptidoglycan is laid down during septation it has a ring conformation which then matures as the cell expands prior to the next round of cell division, and the nascent septum bisects the previous orthogonal “rib” (Figure 6A). Although the AFM images show increased peptidoglycan thickness and surface “roughness” in ECg-grown EMRSA-16 producing a “knobble” texture (Figure 6B) associated with autolysin-mediated remodelling of nascent cell wall ring structures [31], no changes in the mode of peptidoglycan biosynthesis and septum formation are evident (Figure 6B).

## 3. Discussion

Exposure of EMRSA-16 to moderate concentrations of ECg results in rapid insertion of the amphipathic molecule into the CM, producing an initial reduction in the fluidity of the bilayer and induction of the cell wall stress response [23]. The bacteria attempt to compensate for the chemical insult by reconfiguration of the phospholipid composition of the bilayer over a 4 h period during which the growth rate does not change significantly compared to control cultures [23,30]. A progressive increase in CM fluidity due to substitution of unbranched fatty acids with a high proportion of unbranched chains within phospholipids results in a membrane that is significantly more mobile than that of controls. This overcompensation is accompanied by reduced MprF-mediated transfer of lysine residues onto PG head groups, leading to a large reduction (52% to 28%) in LPG and a concomitant increase (35% to 62%) in PG content of the bilayer [23]. Together with a large reduction in the degree of a d-alanyl esterification of cell wall teichoic acid [34], exposure to ECg results in major changes to the overall electrochemical charge profile of the staphylococcal surface (Figure 2). Thus, insertion of ECg into the lipid palisade of the EMRSA-16 CM results in a suboptimal environment in which membrane proteins are forced to operate and this is likely to account for the observed disruption of protein secretion [42] and loss of capacity to form biofilms [35].

ECg was found to displace a substantial proportion of PBP2 from the septal division site [23] and styrene-co-maleic acid extraction of membrane proteins from ECg-grown cells indicated partial physical separation of PBP2 and PBP2a [29]. ECg-grown EMRSA-16 cells also fail to direct a large proportion of autolytic enzymes to sites of ordered peptidoglycan remodeling during the division cycle [35], prompting us to speculate that ECg may disrupt the normal equatorial mode of staphylococcal division in sequential, orthogonal planes [31]. In precedence, it has been determined that dispersal of the staphylococcal cell wall synthetic machinery results in initiation of peptidoglycan synthesis in patches over the entire bacterial surface [43]. AFM revealed this not to be the case for ECg-exposed cells: although examination of peptidoglycan sacculi showed that ECg induced thickened cell walls, it was clear that the most recent division planes occurred perpendicular to previous planes and that extensive remodeling of peptidoglycan occurred between division cycles (Figure 6). ECg-exposed cells are noticeably larger and separate less readily than their normally-grown counterparts (Figure 1B,C) but this is probably due to changes in the distribution of autolysins during cell division [35].

In agreement with Bayer and coworkers [22,24], we found the staphylococcal CM to be highly asymmetric with respect to the distribution of phospholipids between the inner and outer leaflets. These investigators examined a methicillin-susceptible *S. aureus* and a mutant resistant to cationic antimicrobial peptides (CAP), as well an isogenic set of *S. aureus* isolates from the bloodstream of a patient undergoing daptomycin therapy where the isolates were progressively more resistant to daptomycin. They found both CAP and daptomycin resistance to be correlated with small differences in the distribution of LPG; in both cases resistance was linked to a net increase in CM surface charge resulting from a higher proportion of LPG on the outer surface of the membrane. We found the bulk of LPG (95%–97%) in the inner leaflet and only a small proportion in the outer leaflet. PG and CL were located primarily in the outer leaflet (~90% and ~80%, respectively). These proportions are remarkably similar to those obtained by the Bayer group. Intercalation of ECg into the CM had a modest impact on lipid distribution; a significantly increased proportion of PG was located in the inner leaflet after ECg exposure but this is likely to reflect the reduced capacity of MprF to attach lysine residues to PG head groups on the inner surface of the staphylococcal bilayer. The charge profile at the surface does not prevent the rapid incorporation of ECg into the lipid palisade of the staphylococcal CM [23,30] or into artificial lipid vesicles containing the three predominant staphylococcal phospholipids in their natural ratios [30]. The major reconfiguration of the CM induced by ECg intercalation does not include a significant rebalancing of lipid distribution across the bilayer, suggesting that preservation of the degree of lipid asymmetry in crucial to cell viability. This is in marked contrast to the response of many eukaryotic cells to chemical and biological insults, where bilayer imbalances may invoke rapid transfer of positively charged lipid species from the inner to the outer surface of the plasma membrane [44].

There is strong evidence that ECg-mediated sensitization of MRSA to β-lactam antibiotics, as well as other elements of the complex phenotype induced by catechin gallates, is due to functional disruption of proteins within the CM. ECg partially delocalizes PBP2 from the septum of actively dividing cells [23] and elicits physical separation of PBP2 and PBP2a [29]. Beta-lactams are unable to gain access to the active site of PBP2a due to the closed conformation of this domain [45]. We have established [23] that ECg does not directly interact with PBP2a and is therefore very unlikely to induce reversible conformational changes to PBP2a fine structure that could account for the high degree of sensitization to β-lactam antibiotics. As reconfiguration of the bilayer over the 4 h period following the initial ECg exposure does not reverse β-lactam sensitization, it must be considered unlikely that the fluid status of the membrane is the primary determinant of this phenotypic trait, even though ECg intercalation into the lipid palisade engenders a suboptimal environment in which membrane-embedded proteins can function. PBP2 molecules are recruited to the septal division site by a process requiring the presence of FtsZ, a protein that directly or indirectly anchors the cell wall synthetic machinery to the site of cell division, and are retained at this site by pentapeptide substrate interactions [46]. ECg may delocalize PBP2 and disrupt divisome integrity by interfering in some way with this equilibrium.

## 4. Experimental Section

### 4.1. Bacteria

Epidemic MRSA isolate EMRSA-16 was isolated from a clinical sample obtained at the Royal Free Hospital, London. Bacteria were grown at 37 °C in an orbital incubator (200 orbits min) in Mueller-Hinton (MH) broth (Oxoid, Basingstoke, UK) and cells harvested by centrifugation at OD_600_ 0.7. When required, growth medium was supplemented with ECg, provided by Mitsui Norin Co., Tokyo, Japan. ECg was dissolved in 50% *v*/*v* ethanol and added to the bacterial culture to a final concentration of 12.5 µg/mL. MICs were determined by the CLSI broth microplate assay as previously described [32]. Scanning electron microscopy (SEM) of bacteria was undertaken as reported [34]. Net bacterial surface charge was evaluated by determination of binding of equine heart cytochrome c as described by Mukhopadhyay *et al.* [22].

### 4.2. Bilayer Distribution of LPG

Fluorescamine (Sigma Aldrich, Gillingham, Dorset, UK) reacts with primary amines to form a fluorescent product [47] and was used to specifically label the membrane surface-exposed lysine residues on the LPG head group. Following separation of extracted phospholipids by two-dimensional thin layer chromatography (2D-TLC), unlabelled (inner leaflet) LPG can be identified by ninhydrin staining [22]. A solution of 0.52 M fluorescamine in dehydrated dimethylsulfoxide was prepared immediately prior to each experiment. Harvested EMRSA-16 cells were washed once with buffer A (100 mM KH_2_PO_4_, 5 mM EDTA; pH 7.2) and once with buffer B (100 mM KH_2_PO_4_, 600 mM KCl; pH 8.2). Cells (~0.6 g wet weight) were suspended in 3 mL buffer B, transferred to a 25 mL glass conical flask and cooled to 4 °C with gentle mixing; 90 μL fluorescamine was then added drop-wise with constant mixing. After 30 s, the reaction was stopped by addition of 3 mL 1 M NH_3_(aq) in 600 mM KCI. The suspension was centrifuged and the pellet washed with buffer C (200 mM CH_3_COOK, 600 mM KCl; pH 4.5) at 4 °C until the supernatant was free of colour. Phospholipids were extracted from the pellet by vigorous shaking with 5 mL 2:1 *v*/*v* chloroform/methanol, followed by one wash with 4 mL 0.9% NaCl to remove non-lipid contaminants. The organic layer was evaporated to dryness under N_2_ and stored at −20 °C prior to analysis. Phospholipids were separated by 2D-TLC using Silica 60 F254 HPTLC plates (Merck, Darmstadt, Germany). Plates were developed with chloroform/methanol/25% ammonium hydroxide (65:25:6, by vol.) in the first dimension, dried for 30 min and subjected to chloroform/acetone/acetic acid/methanol/water (45:16:9:8:4, by vol.) for separation in the second dimension. Fluorescamine-labelled outer leaflet LPG was detected with a UV detector (excitation at 365 nm) and inner leaflet LPG by ninhydrin staining. PG and CL were identified with molybdenum blue. The spot size of each isolated phospholipid was quantified using PDQuest Advanced 2D Analysis Software (Bio-Rad, Hemel Hempstead, UK). The content of LPG in each cell membrane leaflet was expressed as a percentage of total membrane LPG and the amount of each major phospholipid as the percentage of total phospholipid. Assays were performed a minimum of five times on separate days.

### 4.3. Bilayer Distribution of PG

Two highly fluorescent phospholipid conjugates (Figure S1), obtained from Avanti Polar Lipids Inc. (Alabaster, AL, USA), were employed to investigate PG asymmetry: 1-hexanoyl-2-{6-[(7-nitro-2-1,3-benzoxadiazol-4-yl)amino]hexanoyl}-*sn*-glycero-3-[phospho-*rac*-(1-glycerol)] (06:0–06:0 NBD-PG) and 1-oleoyl-2-{6-[(7-nitro-2-1,3-benzoxadiazol-4-yl)amino]hexanoyl}-*sn*-glycero-3-[phospho-*rac*-(1-glycerol)] (18:1–06:0 NBD-PG). Equilibration of NBD-labelled phospholipids between the two leaflets of the membrane closely resembles the partition of the naturally occurring lipid species [17,38,39]; uptake of NBD label can be quantified by flow cytometry [22] and outer leaflet NBD fluorescence quenched with sodium dithionite [38] to determine distribution across the bilayer. Assay conditions were optimized with respect to bacterial cell density, NBD probe concentration, membrane labelling time and temperature. A 10 mM solution of each fluorescent phospholipid was prepared in DMSO and aliquots stored at −20 °C until required; freshly diluted lipid was prepared for each experiment. NBD-labeled phospholipids (0.1–10 µM) were added to EMRSA-16 cells (10^6^–10^7^ CFU/mL) suspended in PBS and the probes incorporated into the CM at either room temperature or 4 °C. The degree of probe incorporation was monitored at 10 min intervals by flow cytometry. Sodium dithionite (25 mM) was then added and changes in fluorescence emission measured by flow cytometry; unquenched fluorescence corresponded to inner leaflet phospholipid. It is imperative that solutions of 1 M sodium dithionite are freshly prepared in 100 mM Tris pH 9.0 prior to each experiment and stored for no longer than 2 h at 4 °C.

### 4.4. Bilayer Distribution of CL

Two methods were used to determine CL distribution. The fluorescent conjugate 1,1′,2,2′-tetraoleoyl cardiolipin[6-(NBD)aminocaproyl)] (ammonium salt) (18:1–18:1 NBD-CL; Figure S1) was employed in a dithionite quenching assay as described above. In addition, CL asymmetry was investigated using the fluorescent dye 10-*N* nonyl acridine orange (NAO), essentially as described by Mukhopadhyay and coworkers [22]. Fluorescence emission of monomeric NAO peaks at 525 nm. Two molecules of NAO bind to the two phosphate groups of CL and in this dimeric form peak fluorescence emission shifts 640 nm [48]. The dye binds initially to phosphate groups in the outer leaflet of the membrane and binds only to the inner leaflet after saturation of the outer surface; binding dynamics can be used to assess the distribution of adjacent phosphates on CL head groups [48,49]. A 10 mM solution of NAO was prepared in DMSO and stored in aliquots at −20 °C; the dye was diluted to the required concentration immediately prior to use. The impact of cell density, probe concentration and labelling time was investigated to define appropriate conditions for determination of CL asymmetry. Fluorescence emission was determined by flow cytometry.

### 4.5. Flow Cytometry

Fluorescence signals emitted by NBD-phospholipids and NAO were detected using a MACSQuant flow cytometer (Miltenyi Biotec, Bergisch Gladbach, Germany) with excitation at 488 nm, emission at 525 ± 50 nm (green filter) or 655LP-730 nm (red filter). The voltage was set between 300 and 400 V and 10,000 events were acquired at a flow rate of approximately 200 events/s. Unstained control samples were used to locate bacterial populations in forward (FSC) and side (SSC) scatter channels. The acquisition trigger was set using side scatter and the trigger level adjusted to minimize electronic noise. Assays were performed a minimum of five times on separate days.

### 4.6. Confocal Laser Scanning Microscopy (CLSM)

EMRSA-16 cells were labelled with NBD-phospholipids (1–10 µM; labelling time 30–60 min), NAO (0.1–10 µM; 30 min) and Hoechst 33342 (Sigma-Aldrich, Gillingham, Dorset, UK 1–10 µM; 10 min); viability over these concentration ranges was determined by culture on to Mueller-Hinton agar plates. Procedures were conducted in the dark at room temperature. After labelling, cells were washed twice in PBS, fixed (0.42 mL 15% *v*/*v* formaldehyde, 2.08 mL PBS, 0.5 μL 25% *v*/*v* glutaraldehyde) and washed again in PBS. For imaging, cells were mounted on poly-l-lysine-coated slides and examined using a Zeiss LSM 710 confocal microscope (Zeiss International, Oberkochen, Germany) equipped with a 25 mW Argon ion laser (NBD-phospholipids: excitation 488 nm, emission 530 ± 30 nm; NAO: excitation 488 nm, emission 525 ± 40 and 670 ± 60 nm; Hoechst 33342 excitation 405 nm, emission 440 ± 40 nm).

### 4.7. Atomic Force Microscopy (AFM)

The procedure for preparation of EMRSA-16 murein was adapted from Turner *et al*. [31]. A culture volume of EMRSA-16 was collected. Cells from 500 mL MH broth were boiled in a water bath for 7 min to avoid peptidoglycan autolysis and disrupted eight times with 0.1 mm glass beads using a FastPrep instrument (Qbiogene, Carlsbad, CA, USA) for 45 s at speed setting 6. The broken cells were boiled in 5% SDS for 25 min and for a further 15 min in 4% SDS. Wall material was washed with hot distilled water until free of SDS and incubated in Tris-HCL (50 mM, pH 7) with 2 mg/mL pronase at 60 °C for 90 min to remove covalently attached proteins. After a single wash with distilled water, teichoic acids were released from peptidoglycan by incubation in 250 μL hydrofluoric acid for 48 h at 4 °C. The pellets were washed in water until the attained pH ~5 and stored in water at −20 °C. Immediately prior to imaging, flocculates were disrupted by three rounds of sonication (30 s each) and samples diluted in pure water to an appropriate working concentration. Sacculi were loaded onto freshly cleaved mica and dried under a stream of N_2_. As necessary, the washing and drying cycle was repeated to provide enhanced image quality. AFM was carried out in “Tapping Mode” using silicon tips (Olympus, Tokyo, Japan) under ambient conditions using a Multimode or Dimension AFM with an Extended Nanoscope IIIa controller (Veeco Instruments, Plainview, NY, USA). Images were line-corrected using the “Level rows using intersections of given lines” function, flattened by plane subtraction and scales set for optimal contrast in Gwyddion (version 2.8 or greater, Czech Metrology Institute, Brno, Czech Republic).

## 5. Conclusions

ECg intercalates in stable fashion into the cytoplasmic membrane of *S. aureus*. The membrane was found to be highly asymmetric, with LPG predominantly in the inner leaflet and PG and CL predominantly in the outer leaflet. Although ECg induces changes to the fatty acid components of these lipids and reduces the amount of LPG in the bilayer, it has only a small but significant impact on the degree of lipid symmetry. The cells continue to divide in orthogonal mode. Thus, membrane lipid asymmetry and the planes of cell division are preserved during ECg-induced stress.

## Supplementary Materials

Supplementary materials can be found at http://www.mdpi.com/1422-0067/16/08/16710/s1.

## References

[1] Lingwood D., Simons K. (2010). Lipid rafts as a membrane-organizing principle. Science.

[2] Cross T.A., Murray D.T., Watts A. (2013). Helical membrane protein conformations and their environment. Eur. J. Biophys..

[3] Michel V., Bakovic M. (2007). Lipid rafts in health and disease. Biol. Cell.

[4] Van Meer G., Voelker D.R., Feigenson G.W. (2008). Membrane lipids: Where they are and how they behave. Nat. Rev. Mol. Cell Biol.

[5] Fadeel B., Xue D. (2009). The ins and outs of phospholipid asymmetry in the plasma membrane: roles in health and disease. Crit. Rev. Biochem. Mol. Biol..

[6] Venegas V., Zhou Z. (2007). Two alternative mechanisms that regulate the presentation of apoptotic cell engulfment signal in *Caenorhabditis elegans*. Mol. Biol. Cell.

[7] Daleke D.L. (2008). Regulation of phospholipid asymmetry in the erythrocyte membrane. Curr. Opin. Hematol..

[8] Schoenwaelder S.M., Yuan Y., Josefsson E.C., White M.J., Yao Y., Mason K.D., O’Reilly L.A., Henley K.J., Ono A., Hsiao S. (2009). Two distinct pathways regulate platelet phosphatidylserine exposure and procoagulant function. Blood.

[9] Lhermusier T., Chap H., Payrastre B. (2011). Platelet membrane phospholipid asymmetry: From the characterization of a scramblase activity to the identification of an essential protein mutated in Scott syndrome. J. Thromb. Haemost..

[10] Mileykovskaya E., Dowhan W. (2000). Visualization of phospholipid domains in *Escherichia coli* by using the cardiolipin-specific fluorescent dye 10-*N*-nonyl acridine orange. J. Bacteriol..

[11] Kawai F., Shoda M., Harashima R., Sadaie Y., Hara H., Matsumoto K. (2004). Cardiolipin domains in *Bacillus subtilis* marburg membranes. J. Bacteriol..

[12] Barák I., Muchová K., Wilkinson A.J., O'Toole P.J., Pavlendová N. (2008). Lipid spirals in *Bacillus subtilis* and their role in cell division. Mol. Microbiol..

[13] López D., Kolter R. (2010). Functional microdomains in bacterial membranes. Genes Dev..

[14] Bogdanov M., Dowhan W. (2012). Lipid-dependent generation of dual topology for a membrane protein. J. Biol. Chem..

[15] Malinverni J.C., Silhavy T.J. (2009). An ABC transport system that maintains lipid asymmetry in the Gram-negative outer membrane. Proc. Natl. Acad. Sci. USA.

[16] Norris V., Misevic G., Delosme J.M., Oshima A. (2002). Hypothesis: A phospholipid translocase couples lateral and transverse bilayer asymmetries in dividing bacteria. J. Mol. Biol..

[17] Kol M.A., de Kroon A.I.P.M., Killian J.A., de Kruijff B. (2004). Transbilayer movement of phospholipids in biogenic membranes. Biochemistry.

[18] Benfield T., Espersen F., Frimodt-Møller N., Jensen A.G., Larsen A.R., Pallesen L.V., Skov R., Westh H., Skinhøj P. (2007). Increasing incidence but decreasing in-hospital mortality of adult *Staphylococcus aureus* bacteraemia between 1981 and 2000. Clin. Microbiol. Infect..

[19] Thwaites G.E., Edgeworth J.D., Gkrania-Klotsas E., Kirby A., Tilley R., Török M.E., Walker S., Wertheim H.F., Wilson P., Llewelyn M.J. (2011). Clinical management of *Staphylococcus aureus* bacteraemia. Lancet Infect. Dis..

[20] Strommenger B., Bartels M.D., Kurt K., Layer F., Rohde S.M., Boye K., Westh H., Witte W., de Lencastre H., Nübel U. (2014). Evolution of methicillin-resistant *Staphylococcus aureus* towards increasing resistance. J. Antimicrob. Chemother..

[21] Short S.A., White D.C. (1971). Metabolism of phosphatidylglycerol, lysylphosphatidylglycerol, and cardiolipin of *Staphylococcus aureus*. J. Bacteriol..

[22] Mukhopadhyay K., Whitmire W., Xiong Y.Q., Molden J., Jones T., Peschel A., Staubitz P., Adler-Moore J., McNamara P.J., Proctor R.A. (2007). *In vitro* susceptibility of *Staphylococcus aureus* to thrombin-induced platelet microbicidal protein-1 (tPMP-1) is influenced by cell membrane phospholipid composition and asymmetry. Microbiology.

[23] Bernal P., Lemaire S., Pinho M.G., Mobashery S., Hinds J., Taylor P.W. (2010). Insertion of epicatechin gallate into the cytoplasmic membrane of methicillin-resistant *Staphylococcus aureus* disrupts penicillin-binding protein (PBP) 2a-mediated β-lactam resistance by delocalizing PBP2. J. Biol. Chem..

[24] Jones T., Yeaman M.R., Sakoulas G., Yang S.J., Proctor R.A., Sahl H.G., Schrenzel J., Xiong Y.Q., Bayer A.S. (2008). Failures in clinical treatment of *Staphylococcus aureus* infection with daptomycin are associated with alterations in surface charge, membrane phospholipid asymmetry, and drug binding. Antimicrob. Agents Chemother..

[25] Fuda C.C.S., Fisher J.F., Mobashery S. (2005). β-lactam resistance in *Staphylococcus aureus*: The adaptive resistance of a plastic genome. Cell Mol. Life Sci..

[26] Paterson G.K., Harrison E.M., Holmes M.A. (2014). The emergence of *mecC* methicillin-resistant *Staphylococcus aureus*. Trends Microbiol..

[27] Egan A.J.F., Vollmer W. (2013). The physiology of bacterial cell division. Ann. N. Y. Acad. Sci..

[28] Taylor P.W., Hamilton-Miller J.M.T., Stapleton P.D. (2005). Antimicrobial properties of green tea catechins. Food Sci. Technol. Bull..

[29] Paulin S., Jamshad M., Dafforn T.R., Garcia-Lara J., Foster S.J., Galley N.F., Roper D.I., Rosado H., Taylor P.W. (2014). Surfactant-free purification of membrane protein complexes from bacteria: Application to the staphylococcal penicillin-binding protein complex PBP2/PBP2a. Nanotechnology.

[30] Palacios L., Rosado H., Micol V., Rosato A.E., Bernal P., Arroyo R., Grounds H., Anderson J.C., Stabler R.A., Taylor P.W. (2014). Staphylococcal phenotypes induced by naturally occurring and synthetic membrane-interactive polyphenolic β-lactam resistance modifiers. PLoS ONE.

[31] Turner R.D., Ratcliffe E.C., Wheeler R., Golestanian R., Hobbs J.K., Foster S.J. (2010). Peptidoglycan architecture can specify division planes in *Staphylococcus aureus*. Nat. Commun..

[32] Stapleton P.D., Shah S., Anderson J.C., Hara Y., Hamilton-Miller J.M.T., Taylor P.W. (2004). Modulation of β-lactam resistance in *Staphylococcus aureus* by catechins and gallates. Int. J. Antimicrob. Agents.

[33] Brown D.F., Edwards D.I., Hawkey P.M., Morrison D., Ridgway G.L., Towner K.J., Wren M.W. (2005). Guidelines for the laboratory diagnosis and susceptibility testing of methicillin-resistant *Staphylococcus aureus* (MRSA). J. Antimicrob. Chemother..

[34] Bernal P., Zloh M., Taylor P.W. (2009). Disruption of d-alanyl esterification of *Staphylococcus aureus* cell wall teichoic acid by the β-lactam resistance modifier (−)-epicatechin gallate. J. Antimicrob. Chemother..

[35] Stapleton P.D., Shah S., Ehlert K., Hara Y., Taylor P.W. (2007). The β-lactam-resistance modifier (−)-epicatechin gallate alters the architecture of the cell wall of *Staphylococcus aureus*. Microbiology.

[36] Pomorski T., Muller P., Zimmermann B., Burger K., Devaux P.F., Herrmann A. (1996). Transbilayer movement of fluorescent and spin-labeled phospholipids in the plasma membrane of human fibroblasts: A quantitative approach. J. Cell Sci..

[37] Devaux P.F., Fellmann P., Hervé P. (2002). Investigation on lipid asymmetry using lipid probes: Comparison between spin-labeled lipids and fluorescent lipids. Chem. Phys. Lipids.

[38] Angeletti C., Nichols J.W. (1998). Dithionite quenching rate measurement of the inside-outside membrane bilayer distribution of 7-nitrobenz-2-oxa-1,3-diazol-4-yl-labeled phospholipids. Biochemistry.

[39] Dekkers D.W., Comfurius P., van Gool R.G., Bevers E.M., Zwaal R.F. (2000). Multidrug resistance protein 1 regulates lipid asymmetry in erythrocyte membranes. Biochem. J..

[40] Pomorski T., Hrafnsdóttir S., Devaux P.F., van Meer G. (2001). Lipid distribution and transport across cellular membranes. Semin. Cell Dev. Biol..

[41] Bayer A.S., McNamara P., Yeaman M.R., Lucindo N., Jones T., Cheung A.L., Sahl H.G., Proctor R.A. (2006). Transposon disruption of the complex I NADH oxidoreductase gene (*snoD*) in *Staphylococcus aureus* is associated with reduced susceptibility to the microbicidal activity of thrombin-induced platelet microbicidal protein 1. J. Bacteriol..

[42] Shah S., Stapleton P.D., Taylor P.W. (2008). The polyphenol (–)-epicatechin gallate disrupts the secretion of virulence-related proteins by *Staphylococcus aureus*. Lett. Appl. Microbiol..

[43] Pinho M.G., Errington J. (2003). Dispersed mode of *Staphylococcus aureus* cell wall synthesis in the absence of the division machinery. Mol. Microbiol..

[44] Balasubramanian K., Schroit A.J. (2003). Aminophospholipid asymmetry: A matter of life and death. Annu. Rev. Physiol..

[45] Fuda C., Hesek D., Lee M., Morio K., Nowak T., Mobashery S. (2005). Activation for catalysis of penicillin-binding protein 2a from methicillin-resistant *Staphylococcus aureus* by bacterial cell wall. J. Am. Chem. Soc..

[46] Pinho M.G., Errington J. (2005). Recruitment of penicillin-binding protein PBP2 to the division site of *Staphylococcus aureus* is dependent on its transpeptidation substrates. Mol. Microbiol..

[47] Balasubramanian K., Gupta C.M. (1996). Transbilayer phosphatidylethanolamine movements in yeast plasma membranes. Evidence for protein-mediated, energy-dependent mechanism. Eur. J. Biochem..

[48] Petit J.M., Maftah A., Ratinaud M.H., Julien R. (1992). 10 *N*-nonyl acridine orange interacts with cardiolipin and allows the quantification of this phospholipid in isolated mitochondria. Eur. J. Biochem..

[49] Gallet P.F., Petit J.M., Maftah A., Zachowski A., Julien R. (1997). Asymmetrical distribution of cardiolipin in yeast inner mitochondrial membrane triggered by carbon catabolite repression. Biochem. J..

