# Supplementary material for: Impact of the β-Lactam Resistance Modifier (−)-Epicatechin Gallate on the Non-Random Distribution of Phospholipids across the Cytoplasmic Membrane of Staphylococcus aureus
Source: Int J Mol Sci. 2015 Jul 23;16(8):16710–27. doi: 10.3390/ijms160816710 (PMC4581164; doi:10.3390/ijms160816710)
Supplement: Supplementary file 1 [file ijms-16-16710-s001.pdf]

Supplementary Information

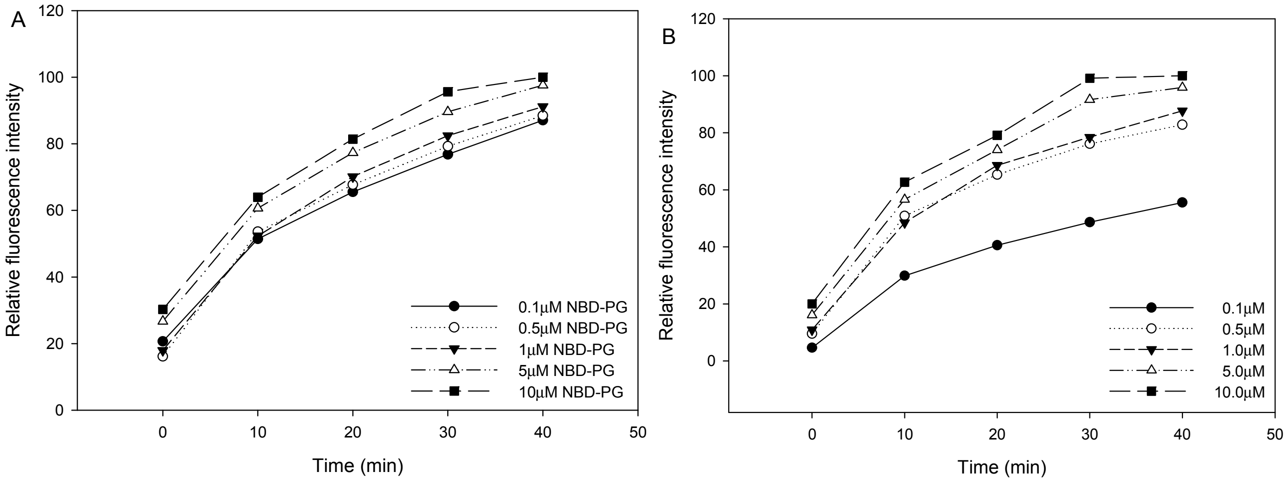

Figure S1. Intercalation of 18:1–06:0 NBD-PG into EMRSA-16 cell membrane at cell densities of 10^6 CFU/mL (**A**) and 10^7 CFU/mL (**B**).

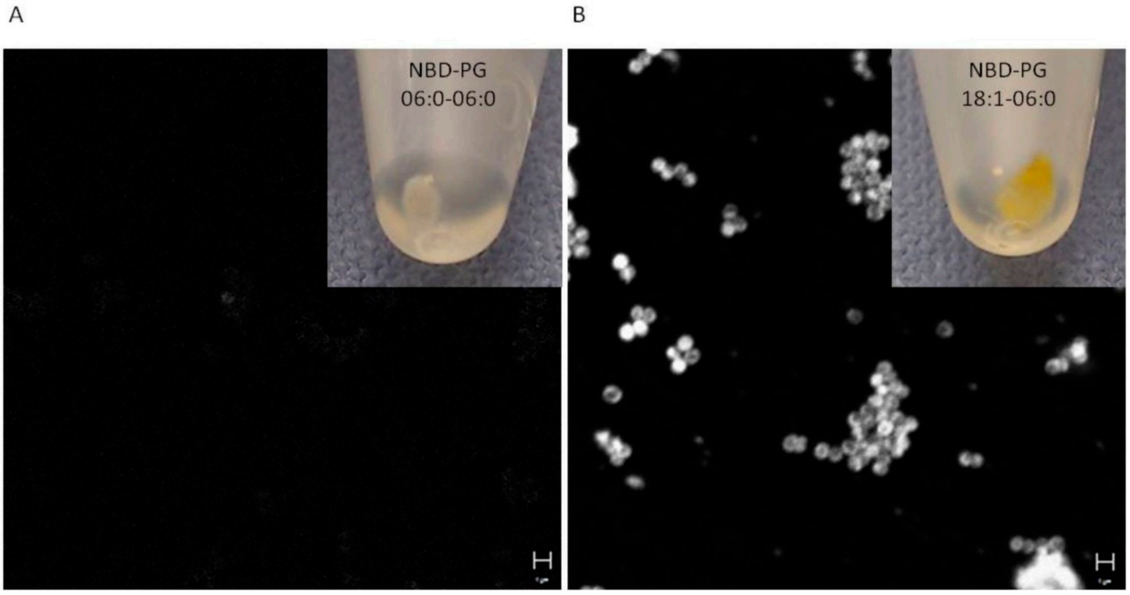

Figure S2. Confocal laser scanning microscopy of EMRSA-16 labelled with 10 µM 06:0–06:0 NBD-PG (**A**) and 18:1–06:0 NBD-PG (**B**). Scale bars 1 µm. Insets show labelled cell pellets.

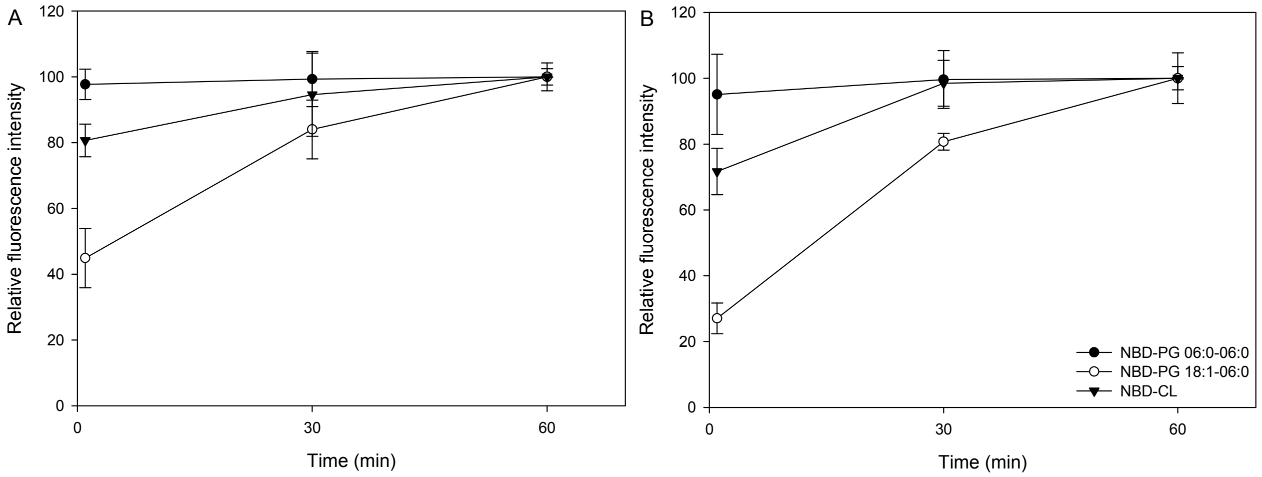

Figure S3. Intercalation of NBD-lipids into the EMRSA-16 membrane in the absence (**A**) or presence (**B**) of 12.5 µg/mL ECg. Fluorescence intensity was measured after incubation with 10 µM of NBD-lipid for 1, 30 and 60 min and the values normalised to the highest fluorescence emission of each NBD-lipid, achieved after 60 min at RT (n = 5; ±1 SD).

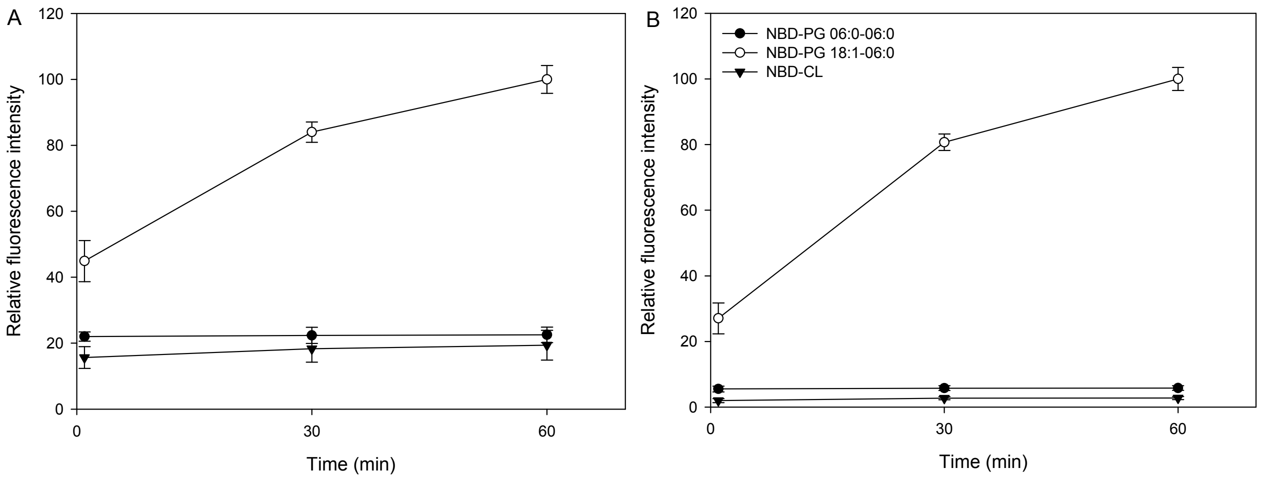

Figure S4. Intercalation of NBD-lipids into the EMRSA-16 membrane in the absence (**A**) or presence (**B**) of 12.5 µg/mL ECg. Fluorescence values were normalized to the highest fluorescence emission signal, achieved with 18:1–06:0 NBD-PG, after 60 min at room temperature (n = 5; ±1 SD).

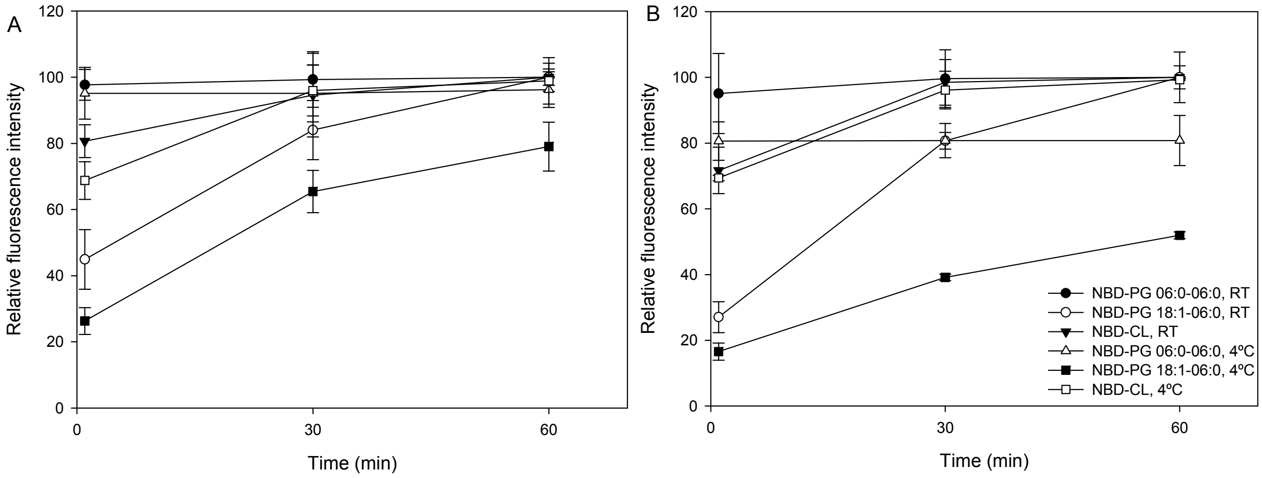

Figure S5. Intercalation of NBD-lipids into the EMRSA-16 membrane at RT and 4 °C in the absence (**A**) or presence (**B**) of 12.5 µg/mL ECg. Fluorescence intensity was determined after incubation with 10 µM of NBD-lipid. Fluorescence values were normalised to the highest fluorescence emission of each NBD-lipid, achieved after 60 min at room temperature (*n* = 5; ±1 SD).

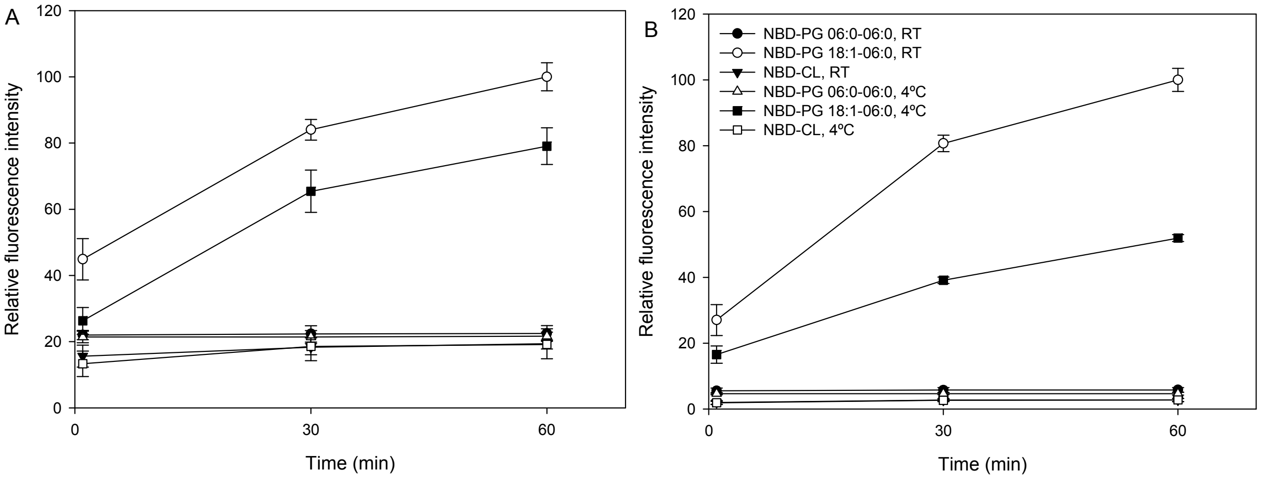

Figure S6. Intercalation of NBD-lipids into the EMRSA-16 membrane at RT and 4 °C in the absence (**A**) or presence (**B**) of 12.5 µg/mL ECg. Fluorescence values were normalized to the highest fluorescence emission signal, achieved with 18:1–06:0 NBD-PG after 60 min at room temperature (*n* = 5; ±1 SD).

A

B

C

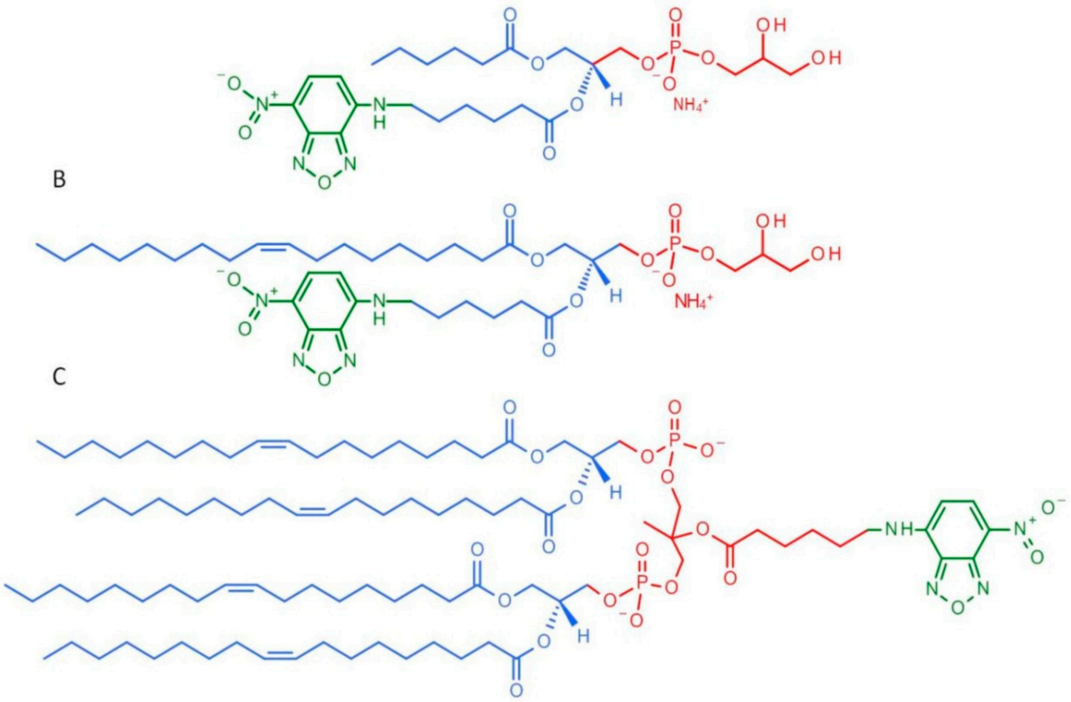

Figure S7. Fluorescent analogues: (**A**) 06:0–06:0 NBD-PG; (**B**) 18:1–06:0 NBD-PG; (**C**) 18:1–18:1 NBD-CL. NBD moieties are highlighted in green, fatty acid chains in blue and head groups in red.
